# Molecular characterization of a rare analphoid supernumerary marker chromosome derived from 7q35 → qter: a case report

**DOI:** 10.1186/s13039-016-0295-z

**Published:** 2016-11-25

**Authors:** Bárbara Marques, Cristina Ferreira, Filomena Brito, Sónia Pedro, Cristina Alves, Teresa Lourenço, Marta Amorim, Hildeberto Correia

**Affiliations:** 1 Unidade de Citogenética, Departamento de Genética Humana, Instituto Nacional de Saúde Doutor Ricardo Jorge, I.P, Avenida Padre Cruz, 1649-016 Lisboa, Portugal; 2 Serviço de Genética Médica, Hospital de Dona Estefânia, Centro Hospitalar de Lisboa Central, Rua Jacinta Marto, 1169-045 Lisboa, Portugal

**Keywords:** Chromosome 7, Analphoid supernumerary marker chromosome, Neocentromere, Partial 7q tetrasomy, 7q duplication

## Abstract

**Background:**

Analphoid supernumerary marker chromosomes (aSMC) constitute one of the smallest groups of SMC, and are characterized by a centromeric constriction but no detectable alpha-satellite DNA. These marker chromosomes cannot be properly identified by conventional banding techniques alone, and molecular cytogenetic methods are necessary for a detailed characterization. Analphoid SMC derived from chromosome 7 are extremely rare, with only five cases reported so far.

**Case presentation:**

In this work we report an aSMC involving the terminal long arm of chromosome 7 in a 10-year-old boy with multiple dysmorphic features and severe development delay. Cytogenetic analysis revealed a mosaic karyotype with the presence of an extra SMC, *de novo*, in 20% of lymphocytes and 73% of fibroblast cells. Next, we performed FISH analysis with multiple DNA probes and cCGH analysis. This identified the origin of the SMC as an analphoid marker resulting of invdup rearrangement of 7q35-qter region.

Affimetrix CytoScan HD array analysis redefined the aSMC as a 15.42 Mb gain at 7q35-q36.3 (minimum tetraplicated region-chr7: 143,594,973-159,119,707; GRCh37/hg19) of maternal origin that encloses 67 OMIM genes, 16 of which associated to disease. Uniparental disomy of chromosome 7 (UPD 7) has been excluded.

**Conclusions:**

We report the first patient with an aSMC(7) derived from the terminal 7q region who has been molecularly and clinically full characterized. The use of SNParray in the characterization of SMC reveals to be a powerful tool, giving information not only about copy number variation but also about loss-of-heterozygosity and parental origin. We conclude that an integrated genome-wide copy number variation analysis, if possible associated to FISH and gene expression studies, could facilitate in the future the difficult task of establishing accurate genotype-phenotype correlations and help to improve genetic counselling.

## Background

Analphoid supernumerary marker chromosomes (aSMC) constitute one of the smallest groups of SMC, and are characterized by a centromeric constriction but no detectable alpha-satellite DNA [1]. These marker chromosomes cannot be clearly identified by conventional banding techniques alone, with molecular cytogenetic methods being necessary for their detailed characterization.

So far 141 aSMC have been reported encompassing 20 of the 22 autossomes and both sexual chromosomes, with 41% of these being acrocentric derivatives (from chromosomes 15 and 13) [2]. The most common aSMC are supernumerary inverted duplications (invdup) of the distal arm of a chromosome. The first aSMC derived from chromosome 7 was reported using multicolour-FISH in a patient with learning and developmental delay as well dysmorphic features [3]. Cases subsequently reported include an inverted-duplication-shaped aSMC derived from the long arm terminal region, and a derivative chromosome 7 with an interstitial deletion of the short and long arms segments showing a neocentromere in the p14 region. None of these reports included the patients’ clinical description [4, 5]. Recently, Kumar et al. reported a child with dysmorphic features and developmental delay, who presented a complex chromosome rearrangement involving chromosome 7 and resulting of a class II/McClintock mechanism [6]. Louvrier et al. [7] reported a mosaic neocentric ring involving the region 7q22.1q31.1, in a child with a severe global retardation and dysmorphic features. In this case, the aSMC characterization, using arrayCGH, and the clinical description are very detailed establishing a clear genotype-phenotype correlation. In our study we used different methods in order to fully characterize an aSMC involving the terminal long arm of chromosome 7 in a patient presenting several dysmorphic features, unstable assisted broad-based walk and severe developmental delay without language acquisition. To our knowledge this is the first case reported of an aSMC(7) with an entire molecular characterization and clinical description involving the region 7q35-qter. With this study we hope to contribute for a better genotype-phenotype correlation and improved genetic counselling.

This case was submitted to the sSMC database (http://ssmc-tl.com/chromosome-7.html#N), No. 07-N-q35/1-1.

## Case presentation

The patient was a newborn male, the first child of a healthy and non-consanguineous couple, born at 28 weeks of gestation with normal somatometric parameters (weight 1167 g corresponding to P50). In the neonatal period the patient showed respiratory distress syndrome and feeding difficulties, and was diagnosed with bronchopulmonary dysplasia probably due to prematurity.

On physical examination, open and wide anterior fontanelle, bilateral eyelid edema, low-set ears, short nose with wide and depressed root, open tented, and fingers and toes nail hypoplasia were described.

The patient also showed bilateral hip dysplasia, cryptorchidism and a bilateral inguinal hernia that has already been corrected. Furthermore, the child was submitted, at 4 months of age to a colectomy for necrotizing enterocolitis, 1 month later to a tracheotomy, and at 18 months of age to a percutaneous endoscopic gastrostomy.

**Fig. 1:**
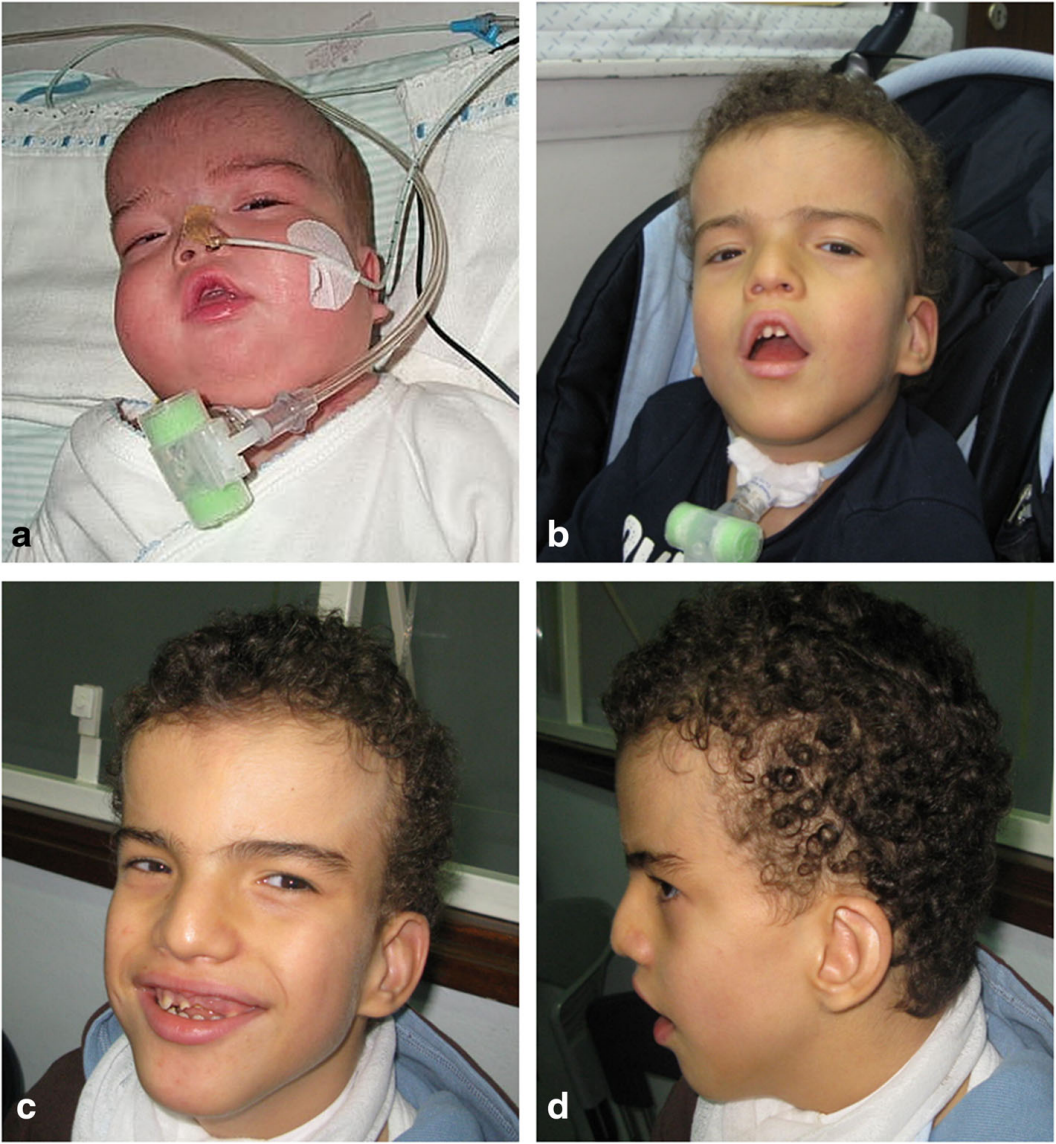
Clinical facial features of the patient at different ages. **a** At 5th months: facial edema, short nose with wide and depressed root; **b** At 4-year-old: turricephaly, curly hair, straight eyebrows with synophrys, opened tented mouth; **c** and **d** At 10-year-old: accentuation of the dysmorphic features with hypertelorism, underdeveloped crus helix, full lips and dental crowding

The patient is currently 10 years old and has severe developmental delay without language acquisition, presenting with bruxism and auto-aggressive behaviour (which started at the age of 8). The child has an unstable, assisted broad-based walk. He still depends on enteral feeding, has a tracheostomy tube for breathing and suffers from recurrent respiratory infections. The physical exam reveals an accentuation of the dysmorphic features with frontal bossing, low-set ears, straight eyebrows with synophrys and hypertelorism. There are also supernumerary teeth, divergent strabismus and anterior chamber asymmetric optic malformation (Fig. 1). Renal and cardiac malformations were excluded by ultrasound.

## Material and methods

### Cytogenetics

Cytogenetic analyses were performed on conventional giemsa-trypsin-leishman (GTL) banded metaphase chromosomes obtained from phytohemagglutinin-stimulated peripheral blood lymphocytes, from the patient and his parents, as well as skin fibroblasts from the patient by standard techniques. A karyotype was established according to the International System for Human Cytogenetic Nomenclature [8].

### Fluorescent *in situ* hybridization (FISH)

FISH analysis was performed on chromosome spreads prepared from lymphocyte and skin fibroblast cultures. FISH probes included alpha-satellite probes for all chromosomes (home made), ELN and D7S427 probes (Oncor, Gaithersburg, MD), whole chromosome painting probe (WCP) for chromosomes 2, 3, 7, 9, 10, 11,16, 17, 19 and 20 (Cambio, Cambridge, UK), subtelomeric 7q probe (TelVysion, Abbott Molecular, Abbott Park, IL, USA) and the bacterial artificial chromosome (BAC) clone RP11-298A10 (kindly supplied by Wellcome Trust Sanger Institute, Cambridge, UK). Extraction of alpha-satellite DNA for FISH probes, labelling and FISH analysis were performed following standard protocols. Extraction of BAC DNA for FISH probe, labelling and FISH analysis were performed as described earlier [9, 10].

### Chromosomal comparative genomic hybridization (cCGH)

Metaphase spreads were prepared from phytohemagglutinin-stimulated lymphocytes from healthy individuals, according to standard procedures. Genomic DNA was extracted from the patient’s peripheral blood sample using the Wizard® genomic DNA purification Kit from Promega (Promega Corporation, WI, USA). cCGH analysis was performed according to standard procedures. The data was analyzed using ISIS-CGH software (MetaSystems, Altleissheim, Germany) with average ratio profiles of 1,25 e 0,75 and standard deviation limits.

### SNParray

Array analysis was performed in genomic DNA extracted from peripheral blood of the patient and his parents using Affymetrix CytoScan HD® array (Affymetrix, California, USA) according to the manufacturer’s recommendations (Affymetrix manual protocol Affymetrix® Cytogenetics Copy Number Assay P / N 703038 Rev. 3). CytoScan HD array contains 740,304 polymorphic (SNP, single nucleotide polymorphism) and 1,953,249 non-polymorphic (copy number probes) markers with an average intragenic marker spacing of 880 bps and intergenic marker spacing of 1737 bps. The raw data were processed using Genotyping Console v4.0 and Chromosome Analysis Suite 3.0.0.42 with NetAffx na33.1 (UCSC GRCh37/hg19) and the output data were interpreted with the UCSC Genome Browser (https://genome.ucsc.edu/; GRCh37/hg19 assembly), DECIPHER (https://decipher.sanger.ac.uk/) and ClinGen (http://www.clinicalgenome.org). The functions of the genes, which were located within the region of the genomic imbalance, were retrieved from the GeneCards (http://www.genecards.org) and OMIM (http://www.ncbi.nlm.nih.gov/omim) databases. The trio analysis to exclude uniparental disomy of chromosome 7 (UPD 7) was done using the CytoScanHD_Array Mendelian Error Check tool and the parental origin of the aSMC was determined using MyPODFinder v.1.0.

## Results

**Fig. 2:**
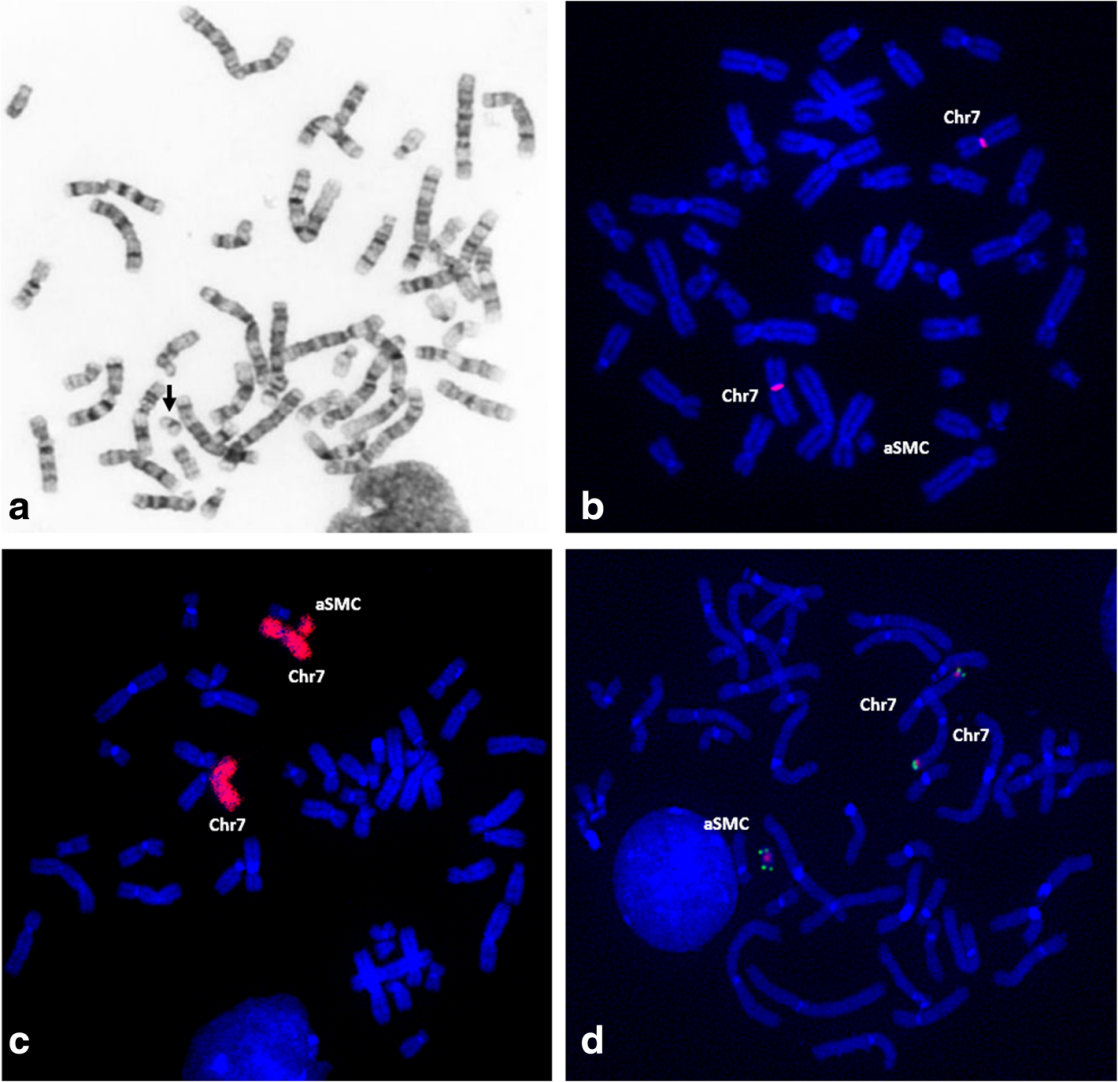
Cytogenetic and FISH investigation **a** GTL-banded chromosomes showing the aSMC (arrow); FISH with **b** α-satellite probe for chromosome 7 showing absence of signal on the aSMC; **c** WCP(7) showing the presence of chromosome 7 euchromatin in the aSMC; **d** BAC RP11-298A10 (*red*) e subtelomeric 7q (*green*) probes showing that the aSMC is a invdup involving the terminal region of chromosome 7 (7q35-qter)

Cytogenetic analysis revealed a mosaic karyotype with the presence of a SMC, *de novo*, in 20% of lymphocytes and 73% of skin fibroblast cells (Fig. 2a).

FISH analysis with alpha-satellite probes for all chromosomes indicated that the SMC was an analphoid marker (Fig. 2b), while the presence of euchromatic material was revealed with whole chromosome painting probe for chromosome 7 (Fig. 2c). cCGH analysis indicated that the euchromatic material had origin in the 7q35-qter region (data not shown). Hybridization with RP11-298A10 and subtelomeric 7q probes, allowed establishing that the aSMC results of an invdup rearrangement of 7q35-qter region (Fig. 2d).

**Fig. 3:**
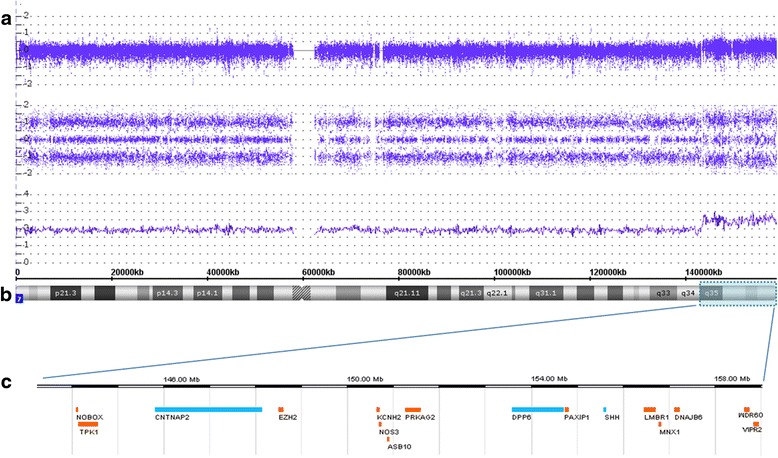
SNParray profile for chromosome 7. **a** Copy number probe intensities (log2 ratio) are represented in the upper track, allele peak tracks in the middle and smooth signal below indicating a mosaic gain. **b** Chromosome 7 ideogram showing the tetraplicated region (*blue* box); **c** OMIM genes associated to disorder present in the aSMC: In *blue* = more potentially implicated in phenotype; In *orange* = apparently not related to phenotype

Affimetrix CytoScan HD array analysis redefines the aSMC to a region of about 15.42 Mb enclosing 67 OMIM genes, 16 of which are associated to disease (Fig. 3). Trio analysis of the patient and his parents excluded the presence of UPD 7 and indicated a maternal origin of the aSMC.

Based on these results, the karyotype was established as: 

mos 47,XY,+mar dn[73]/46,XY[27].ish invdup(7)(qter → q35::q35 → neo → qter)(wcp7+,D7Z1-,RP11-298A10++,TelVysion7q++).arr[GRCh37] 7q35q36.3(143218954x2,143594973_159119707x4).

## Discussion

When an SMC is discovered in a current cytogenetic analysis, its identification can be a challenging task if using FISH only. The best approach to study an SMC with euchromatic material is to use DNA microarray to determine its origin, involved region and size, followed by FISH for its characterisation. In our case, the SMC identification and characterization was made by FISH and cCGH. Afterwards, SNParray was done to mapping at submicroscopic level the genomic imbalance, to exclude the presence of UPD and to determine the parental origin. This allowed the characterization of an aSMC, which consists of an invdup(7) of maternal origin, resulting in tetrasomy of 7q35qter region, with a size of 15.42 Mb and no morbid gene disrupted at the breakpoint. It contains 67 OMIM genes, 16 of which associated to disease and therefore predicted to cause phenotypic effects.

The correlation of syndromic manifestations with specific chromosome segments duplications is not simple, since a pure 7q partial trisomy is rare. In 2008, Scelsa et al. reviewed 18 cases with apparently pure dup(7) and compared them with a patient who had trisomy of 7q32-7qter region [11]. In this study the authors classified the patients into four groups according to the size of the rearrangement. The last group, were we can include our patient, presents distal duplications and the most frequent phenotypic features are: macrocephaly, frontal bossing, small nose, low-set ears and usually severe developmental delay. However, from the seven cases reported in this group, only a tandem duplication of 7q36-qter could be considered as pure dup(7) since it does not involve another chromosome [12]. In the other six cases the duplicated material is present as part of a derivative chromosome that arises from a parent’s balanced translocation, hence the contribution of an eventual positional effect or a gene regulatory disruption to the phenotype cannot be excluded [13–15]. In addition, these cases were not run through genome-wide studies so the presence of a single genomic imbalance region could not be confirmed. DECIPHER database lists 109 patients with a 7q gain that overlaps with our patient, but only 49 have a single region of imbalance [16]. There is only one patient with a small triplicate region of 354.50 Kb [DECIPHER ID 251853: arr[GRCh37] 7q36.3(157930785_158285281)×3] which was inherited from a normal parent, that comprises *PTPRN2* (OMIM*601698), and who presented with broad thumb, delayed speech and language development, hypertelorism and intellectual disability. Furthermore, seven of these patients have copy number gains of more than 1 Mb, being the biggest a *de novo* gain of 11.50 Mb [DECIPHER ID 268243: arr[GRCh37] 7q33q36.1(137914589_149415401)×3] that encompasses several genes, including *CNTNAP2* (OMIM*604569), whilst presenting with developmental delay and strabismus. Intellectual disability, delayed speech and language development are the most common phenotypic features associated with terminal 7q gains, however global development delay and specific learning disability can also be found.

The tetraplicated region of our patient encloses 16 morbid genes. The analysis of these genes permitted to highlight *CNTNAP2* (OMIM*604569), *DPP6* (OMIM*126141) *and SHH* (OMIM*600725). *CNTNAP2* and *SHH* are confirmed as causing developmental disorder in multiple unrelated cases [16]. *CNTNAP2* was linked to complex neurological disorders, including language impairment, autism, intellectual disability, schizophrenia, epilepsy, speech delay, attention deficit hyperactivity disorder, cortical dysplasia-focal epilepsy and Pitt-Hopkins-like syndrome [17–19]. *SHH* (sonic hedgehog) belongs to a family of genes that code for a class of proteins that act as signalling molecules during embryogenesis, namely for nervous and skeletal systems and formation of the testis cord [20]. *DPP6* has been related to microcephaly and mental retardation as well in the pathogenesis of autism spectrum disorder and Gilles de la Tourette syndrome [21–23]. Most of the reports associate the phenotype of patients to haplo-insufficiency of these genes, resulting from deletions, mutations or disruptions, and not to overexpression. However, the overexpression of the *SHH* was suggested to be responsible for the typical facial features and profound hypotonia observed in the distal 7q duplication syndrome [24] and for the congenital muscular hypertrophy described in siblings with a 0.3 Mb duplication at 7q36.3 [25]. Recently, Wong et al. (2015) described a familial 7q36.3 duplication of 0.73 Mb involving *SHH* and *RBM33* associated with agnesia of the corpus callosum, macrocephaly, broad forehead, widely spaced eyes and mild intellectual disability [26]. On the other hand, overexpression of *PRKAG2* has been associated to cardiac problems observed in a case of partial tetrasomy of 7q35q36.3 region [27]. Marshall et al. (2008) performed genome-wide studies in 427 families with autism spectrum disorders (ASDs) using SNParray, and found that both deletions and duplications on *DPP6* are implicated as a novel locus for this disease [23]. On the other hand different expression levels of *LMBR1* (OMIM*605522), required for limb formation, were implicated in acheiropodia (ACHP, OMIM#200500) and triphalangeal thumb-polysyndactyly syndrome (TPTPS, OMIM#174500) [28]. ZRS is a long-range limb-specific *SHH* enhancer, on chromosome 7q36.3 region, which lies within intron 5 of *LMBR1* approximately 1 Mb upstream of the target gene *SHH*, and determining the identity and number of digits in early limb development [29]; duplication of this gene was proposed to originate syndactyly type IV (SD4) and TPTPS as a continuum of phenotypes [30]. Some limb abnormalities can be found in patients with 7q duplications, such as a wide space between the first and second toes [24] campodactyly with 5^th^ finger clinodactyly [13], adducted thumbs [11] and short fifth fingers with clinodactyly [15]. Nevertheless, our patient has 4 copies of *PRKAG2* and *LMBR1* (and ZRS), but does not present any cardiac affectation or alteration in the number or form of fingers and toes, showing only nails hypoplasia. However, little is known about the expression of the genes involved in the tetraplicated region, and the absence of cardiac and limb abnormalities could be explained by the mosaicism and/or a possible dosage compensation mechanism.

The smaller duplication of 7q36qter region described [12] is not associated with any major malformation, but with developmental delay, particularly in speech. It is important to note that this rearrangement was characterized by cytogenetics only, and that *CNTNAP2* gene spans 2.3 Mb, between the 7q35 and 7q36 bands, and is probably disrupted in this case which reinforces the importance of this “language“gene.

## Conclusion

In summary, this is the first patient with an aSMC(7) derived from the terminal 7q region who has been fully characterized by high resolution genome-wide analysis and has also a detailed clinical description. This report confirms that euchromatic supernumerary material from distal region of 7q can cause a severe phenotype with mental retardation and dysmorphisms, being language, particularly speech, the most obviously affected areas of development. This new study contributes to the knowledge of one more aSMC(7), the sixth case reported so far, and to a better understanding of this type of chromosome rearrangements which make genetic counselling extremely challenging.

We reinforce the need for a better characterization of all new findings in this region, using more informative methods, as well as the importance of a detailed clinical description of all the patients registered in databases, in order to allow a better interpretation of genomic variants. An integrated analysis of genome-wide copy number variation and parental origin, if possible associated to FISH and gene expression studies could facilitate in the future the difficult task of establishing accurate phenotype-genotype correlations and aid in genetic counselling.

## Abbreviations

arrayCGH: Array comparative genomic hybridization
ASDs: Autism-spectrum disorders
aSMC: Analphoid supernumerary marker chromosome
BAC: Bacterial artificial chromosome
cCGH: Chromosomal comparative genomic hybridization
FISH: Fluorescent *in situ* hybridization
GTL: Giemsa-trypsin-leishman
SD4: Syndactyly type IV
SMC: Supernumerary marker chromosome
SNParray: Single nucleotide polymorphism array
TPTPS: Triphalangeal thumb-polysyndactyly syndrome
UPD 7: Uniparental disomy of chromosome 7
WCP: Whole chromosome painting probe
